# Editorial: The biochemistry of Mediterranean plants under abiotic stress

**DOI:** 10.3389/fpls.2023.1307316

**Published:** 2023-10-26

**Authors:** Laura Siracusa

**Affiliations:** Istituto di Chimica Biomolecolare del Consiglio Nazionale delle Ricerche (ICB-CNR), Catania, Italy

**Keywords:** biodiversity, specialized metabolism, plant response, abiotic stresses, physiology, strategic crops, Mediterranean area

With reference to the so called ecological pyramid, dealing with the distribution of living organism on their reciprocal relationship basis, plants always lay at its base as they are the sole producers of glucose, the key molecule at the center of biochemical cycles. Among the living organisms, plants can also be considered as those possessing the highest levels of biodiversity, reflecting in a huge chemodiversity in their specialized metabolism (Yonekura-Sakakibara and Saito, 2009; Macel et al., 2010). As a matter of fact, any biological activity attributed to plants is related with their specialized metabolite content; these small molecules were improperly classified as *secondary* metabolites to distinguish them from those defined as primary, such as carbohydrates, proteins, and nucleic acids which are present in every living organism and essential for vital processes. On the contrary, the distribution of secondary metabolites is not ubiquitous but preferential in a genus, a family or even in a particular species. Secondary metabolism is influenced by both genetic and environmental factors (the chemical profile of an essential oil from an aromatic plant is a typical example), and for this reason, thanks to the study of the secondary metabolism of an organism it is possible to acquire information on its origins and its typicality. In recent decades it has been understood that secondary metabolites are as essential for life as primary ones, as they are responsible for the *defense* and communication processes among plants and between plants and other species (insects, ruminants), therefore assuming the role of regulators of intra- and inter-specific relationships (Siracusa and Ruberto, 2014).

By the study of plants’ specialized metabolism researchers may acquire information on:

- chemotaxonomic identification of botanically similar or identical species
- chemodifferentiation (chemotypes)
- chemoconvergence recognition of different species sharing part of secondary
metabolism (parallel evolution)
- identification of the role of primary/secondary hybrid metabolites (eg inositols)
- chemoecology: unravelling the mechanisms of communication and defense in plants

With reference to the last point, which this Research Topic partly deals with, specialized metabolism differ among species also when plants experience a wide variety of stresses, natural or induced. Soil, water and air pollution, climate change, weeds, pathogens are just a few examples of the variegate factors influencing plant metabolism and addressing plant response. In this article Research Topic, the focus has been put on the biochemical response of plants on *abiotic* stresses such as heat, drought, and salinity. The study of how plants interact with the environment and how they activate their defense mechanisms is nowadays of pivotal importance, as the Mediterranean area is experiencing remarkable changes in temperature and humidity, drought, and UV radiation. These changes affect the entire ecosystem, human beings included. In fact, production, quality and resistance of crops to abiotic stresses can severely modify human habit and food consumption directly (vegetable food) or indirectly (animal food and related products). In this scenario, the articles belonging to this Research Topic present some examples of how much variegate the plant response to abiotic stresses could be.

One example is the targeted development of functional traits (FT) in species living in harsch environments with the ultimate aim of survive, as reported by Hidalgo-Triana et al. The authors studied how and at what extent plant FTs react to serpentine soils containing very low nutrients amounts and high levels of heavy metals. Their study was conducted on a broad and variegate collection of plants, counting 24 different species, including woody and perennial herbaceous plants belonging to 14 taxonomic families and 21 genera. All species selected share the common feature of exhibiting morphological-functional adaptations to the Mediterranean dry season. Their original results showed that serpentine ecosystems are characterized by a broad biodiversity, being composed of species with a wide ecological range of functional traits. The authors also demonstrate that physiological changes due to plant response at metabolic level may also be accompanied by macroscopic changes as the development of targeted “survival” traits.

As previously stated, plant response to abiotic stresses is of pivotal importance especially when edible plants are involved, due to their role in human consumption and as animal feed. This is the case of two strategic cereal crops in the Mediterranean area, wheat (*Triticum* spp.) and barley (*Hordeum vulgare*). Wang and Chang reviewed the importance of hydrophobic cuticle biosynthesis and development as first line of defense in these two crops. In fact, wheat and barley show remarkable adverse effect for yield and quality when affected by abiotic stresses such as those primarily considered in this Research Topic (drought, extreme temperatures, and salinity). The authors exhaustively discussed recent strategies, challenges and perspectives on how to manipulate cuticle biosynthesis thus improving wheat and barley resistance. Another crop that has recently gained economic importance in the Mediterranean area is hop (*Humulus lupulus L.*), mainly cultivated for the beer industry but also a precious source of uncommon metabolites (e.g. α and β-acids, and prenylated flavonoids). In their mini-review published in this Research Topic, Marceddu et al. reported the most significant research advances in the cultivation of this species in the Mediterranean area, with particular focus on its responses to the main abiotic stresses and the effects on these stresses on productive and qualitative traits of the plant. The data reviewed by the authors are extremely useful for the identification of functional strategies to boost hops resilience and improve hops cultivation in semi-arid Mediterranean environments. The variegate and multilevel effects of abiotic stresses on whole ecosystems, including plants, insects, and humans is fully described in the original research article proposed by Descamps et al. The authors studied the change in yield and composition of two plant products, nectar and pollen, during spring and summer in temperate regions. These periods are crucial for flowering, communication and reproduction in the majority of plants; furthermore, nectar and pollen are important nutritional resources for pollinators and for humans. The authors demonstrated that, in the bee pollinated species *Borago officinalis*, both nectar and pollen underwent remarkable changes when subjected to climate changes (drought + heatwaves), and discussed these modifications under the double point of view of plant–pollinator interactions and insect nutritional needs. Taken as a whole, the articles presented in this Research Topic provide an exhaustive and variegate point of view on the pivotal importance of monitoring plants’ responses to abiotic stresses such as those inflicted by climate change, offering as the same time a robust prospective on how plants deal with inevitable temperature, salinity, and humidity changes.

## Author contributions

LS: Writing – original draft, Writing – review & editing.
